# Crosstalk between Nuclear Factor I-C and Transforming Growth Factor-β1 Signaling Regulates Odontoblast Differentiation and Homeostasis

**DOI:** 10.1371/journal.pone.0029160

**Published:** 2011-12-16

**Authors:** Dong-Seol Lee, Won-Joon Yoon, Eui Sic Cho, Heung-Joong Kim, Richard M. Gronostajski, Moon-Il Cho, Joo-Cheol Park

**Affiliations:** 1 Department of Oral Histology-Developmental Biology and Dental Research Institute, School of Dentistry, Seoul National University, Seoul, Korea; 2 Department of Molecular Genetics, BK21 Project, School of Dentistry, Seoul National University, Seoul, Korea; 3 Laboratory for Craniofacial Biology, Institute of Oral Bioscience, Chonbuk National University, Jeon-ju, Korea; 4 Department of Oral Anatomy and Developmental Biology, College of Dentistry, Chosun University, Gwang-ju, Korea; 5 Department of Biochemistry, Program in Neuroscience, Developmental Genomics Group and Center of Excellence in Bioinformatics and Life Sciences, School of Medicine and Biomedical Science, State University of New York at Buffalo, Buffalo, New York, United States of America; 6 Department of Oral Biology, School of Dental Medicine, State University of New York at Buffalo, Buffalo, New York, United States of America; The National Institute of Diabetes and Digestive and Kidney Diseases, United States of America

## Abstract

Transforming growth factor-β1 (TGF-β1) signaling plays a key role in vertebrate development, homeostasis, and disease. Nuclear factor I-C (NFI-C) has been implicated in TGF-β1 signaling, extracellular matrix gene transcription, and tooth root development. However, the functional relationship between NFI-C and TGF-β1 signaling remains uncharacterized. The purpose of this study was to identify the molecular interactions between NFI-C and TGF-β1 signaling in mouse odontoblasts. Real-time polymerase chain reaction and western analysis demonstrated that NFI-C expression levels were inversely proportional to levels of TGF-β1 signaling molecules during *in vitro* odontoblast differentiation. Western blot and immunofluorescence results showed that NFI-C was significantly degraded after TGF-β1 addition in odontoblasts, and the formation of the Smad3 complex was essential for NFI-C degradation. Additionally, ubiquitination assay results showed that Smurf1 and Smurf2 induced NFI-C degradation and polyubiquitination in a TGF-β1-dependent manner. Both kinase and *in vitro* binding assays revealed that the interaction between NFI-C and Smurf1/Smurf2 requires the activation of the mitogen-activated protein kinase pathway by TGF-β1. Moreover, degradation of NFI-C induced by TGF-β1 occurred generally in cell types other than odontoblasts in normal human breast epithelial cells. In contrast, NFI-C induced dephosphorylation of p-Smad2/3. These results show that crosstalk between NFI-C and TGF-β1 signaling regulates cell differentiation and homeostatic processes in odontoblasts, which might constitute a common cellular mechanism.

## Introduction

Tooth formation is regulated by sequential and reciprocal epithelial-mesenchymal interactions. Dental epithelial cells from the dental organ differentiate into ameloblasts, while ectomesenchymal cells from the dental papilla differentiate into odontoblasts [1]. Differentiating odontoblasts elongate, polarize, and produce dentin by synthesizing and secreting dentin sialophosphoprotein (DSPP) and collagen type I alpha1 (COLIA1), a marker protein of odontoblasts [2], [3]. An essential role of odontoblasts is the production of a thick dentin layer that forms the bulk of the tooth. However, the molecular mechanisms underlying odontoblast differentiation are not well understood.

The nuclear factor I (NFI) family of site-specific transcription factors, encoded by four genes in vertebrates (i.e., *Nfia*, *Nfib*, *Nfic*, *and Nfix*), plays essential developmental roles in the transcriptional modulation of various cell types [4]. Disruption of the *Nfi* genes in mice leads to developmental defects in brain (*Nfia*) [5], lung and brain (*Nfib*) [6], [7], and brain and skeleton (*Nfix*) [8]. Interestingly, knockout of the NFI-C gene in mice results in tooth defects, including abnormal odontoblast differentiation during molar root formation and short root formation [9].

The transforming growth factor-β (TGF-β) superfamily, including TGF-β, activins, inhibins, and bone morphogenetic protein, regulate cell proliferation, cell differentiation, the epithelial-to-mesenchymal transition, and embryonic development. Several reports have provided evidence supporting the interaction between NFI-C and TGF-β signaling. The NFIC-binding site was found to overlap with the TGF-β response element in the promoter regions of extracellular matrix genes, including rat bone sialoprotein (BSP) [10], the plasminogen activator inhibitor gene [11], and ColIa1 [12]. In addition, experiments searching for DNA-binding domains of Smads and members of the NFI/CAAT box-binding family indicated that they belong to a new superfamily of genes [13], and that Smad3 and Smad4 interact strongly with NFI-C through their MH1 domains [14]. However, the exact mechanism of the interaction between NFI-C and TGF-β signaling remains unclear.

In the present study, we focused on NFI-C and TGF-β1, which are essential factors for both normal odontoblast differentiation and dentin formation. Disruption of these two genes leads to abnormalities in these processes [15], [16]. Therefore, we tested the hypothesis that the balanced interaction between NFI-C and TGF-β1 signaling regulates cellular differentiation and homeostatic processes in odontoblasts.

## Results

### Patterns of gene expression during odontoblast differentiation

**Figure 1:**
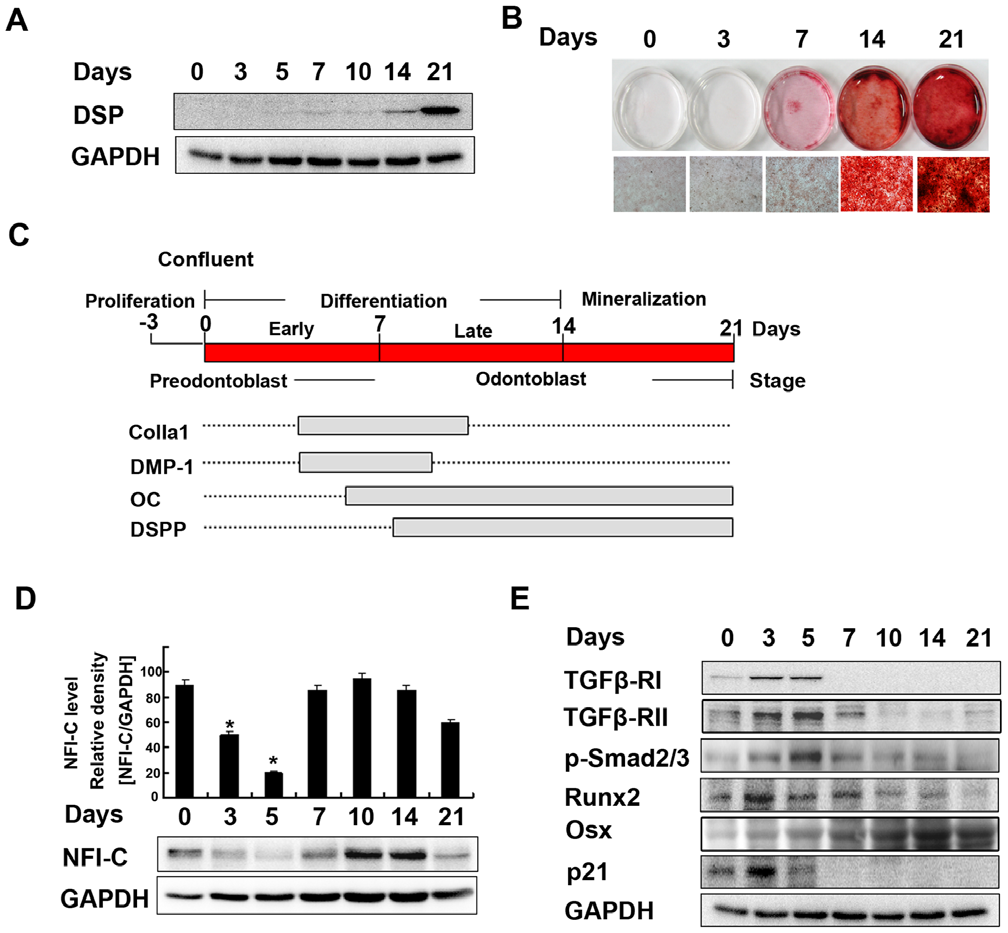
Patterns of gene expression during odontoblast differentiation. MDPC-23 cells were cultured in differentiation medium for up to 3 weeks. (**A**) The expression of DSP was evaluated by western blot analysis. GAPDH was used as a loading control. (**B**) Mineralized nodules stained with alizarin red-S were photographed. (**C**) Four stages of differentiation were identified: confluent (preodontoblast; 0 days); early odontoblast differentiation (∼7 days); late odontoblast differentiation (7∼14 days); and mineralization (14∼21 days). Solid gray bars indicate the periods of elevated expression of genes indicated during culture. (**D**) The expression of NFI-C was evaluated by western blot analysis and the results were quantified using ImageJ. (**E**) TGFβ-RI, TGFβ-RII, p-Smad2/3, Runx2, Osx, and p21 were evaluated by western blot analysis.

MDPC-23 cells are immortalized undifferentiated dental papilla cells that are capable of differentiating into odontoblasts that express DSPP, a dentin-specific gene, and forming mineralized nodules. To determine the stage of odontoblast differentiation *in vitro*, MDPC-23 cells were cultured in differentiation medium for up to 3 weeks and the expression levels of odontoblast differentiation markers were analyzed by western blot and real-time polymerase chain reaction (PCR). The formation of mineralized nodules was evaluated by alizarin red-S staining. The expression levels of *osteocalcin* mRNA gradually increased from days 5 to 14 (Figure S1A). Expression of *DSPP* mRNA, a marker of differentiated odontoblasts, was detected at day 7 and significantly increased by day 21 (Figure S1B), while the expression of dentin sialoprotein (DSP) was detected by western blot on day 14 and continued to increase through day 21 (Figure 1A). Expression of *ColIa1* and *dentin matrix protein 1* (*DMP-1*) mRNA, early marker genes of odontoblast differentiation, increased from days 4 to 7, and then markedly decreased at day 14 (Figure S1C and D). Alizarin red-S staining revealed the presence of mineralized nodules from days 7 to 21 (Figure 1B). Based on these findings, we divided odontoblast differentiation into four stages in MDPC-23 cells: confluent (preodontoblast; day 0); early odontoblast differentiation (days 0∼7); late odontoblast differentiation (days 7∼14); and mineralization (days 14∼21; Figure 1C).

*Nfic*-deficient mice have aberrant odontoblast differentiation and dentin formation during tooth root development and higher levels of TGFβ receptor I (TGFβ-RI) and p-Smad2/3 in their abnormal odontoblasts and pulp cells [15]. Therefore, we next investigated the expression levels of NFI-C and TGF-β signaling molecules during *in vitro* odontoblast differentiation by western blot. NFI-C protein was expressed at the beginning of the culture, decreased from days 3 to 5 (early odontoblast differentiation), increased from days 7 to 14 (late odontoblast differentiation), and then decreased thereafter (Figure 1D). However, the protein level of TGFβ-RI, TGFβ-RII, p-Smad2/3, Runx2, and p21 showed the opposite pattern to that of NFI-C. The expression levels of those five proteins increased from days 3 to 5, and then declined gradually from days 7 to 21, corresponding to late odontoblast differentiation and mineralization (Figure 1E). On the other hand, the protein level of osterix (Osx) increased gradually in both late odontoblast differentiation and mineralization (days 7∼21; Figure 1E).

### TGF-β1 induces NFI-C degradation in odontoblasts

**Figure 2:**
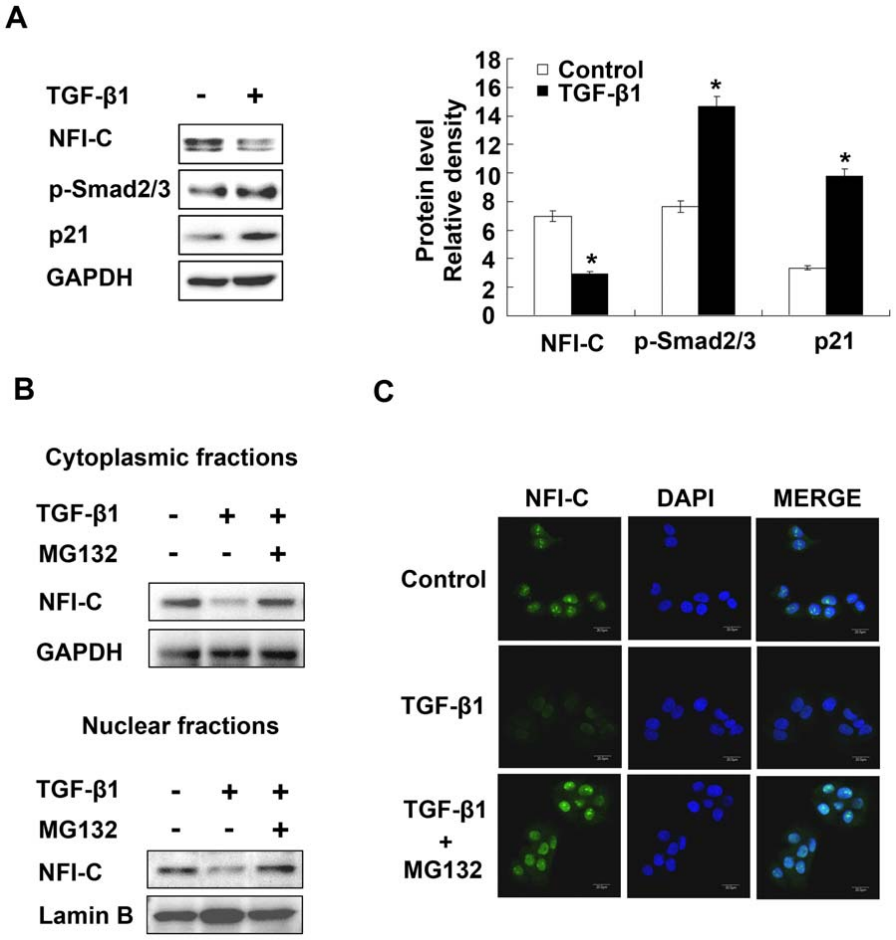
NFI-C is degraded by TGF-β1 in MDPC-23 cells. (**A**) The protein levels of NFI-C, p-Smad2/3, and p21 protein in TGF-β1-treated MDPC-23 cells were analyzed by western blot (left panel), and the results were quantified using ImageJ (right panel). GAPDH used as a loading control. (**B**) MDPC-23 cells were incubated with TGF-β1 (10 ng/ml) in the presence or absence of the proteasome inhibitor, MG132 (10 µM) for 1 hr. Cytoplasmic and nuclear fractions were isolated and subjected to western blot analysis. GAPDH and lamin B served as cell fractionation controls. (**C**) The subcellular localization of NFI-C was analyzed by immunostaining with NFI-C specific antibody. DAPI staining was used to detect nuclei.

During *in vitro* odontoblast differentiation, we noted inverse patterns of expression for NFI-C and TGF-β signaling molecules during early odontoblast differentiation (Figure 1D and E). To determine whether the decrease in NFI-C protein levels observed during early odontoblast differentiation was affected by TGF-β signaling, we measured the effect of TGF-β1, TGF-β2, and TGF-β3 treatment on the level of endogenous NFI-C protein in MDPC-23 cells. TGF-β2 and TGF-β3 hardly influenced the level of NFI-C protein expression but TGF-β1 decreased NFI-C protein levels (Figure S2A). Overexpression of activated TGFβ-RI also significantly decreased NFI-C protein levels (Figure S2B). Interestingly, the levels of NFI-C protein expression were decreased by TGF-β1 in a concentration-dependant manner (Figure S2C). In addition, TGF-β1 increased expression levels of p-Smad2/3 and p21 (Figure 2A).

A number of intracellular proteins are degraded via the proteasome-dependent pathway [17]. To determine whether degradation via the proteasome is involved in the TGF-β1-dependent reduction of NFI-C, we used MG132, a specific inhibitor of the proteasome. Decreased levels of NFI-C were observed in the cytoplasmic and nuclear fractions of MDPC-23 cells in response to TGF-β1 treatment. However, pre-treatment of MDPC-23 cells with 10 µM MG132 for 1 hr prevented TGF-β1-induced NFI-C degradation in both fractions (Figure 2B). Furthermore, NFI-C protein accumulated in the nuclei of MDPC-23 cells after treatment with TGF-β1 and MG132, as observed by immunofluorescence (Figure 2C). These data suggest that the degradation of NFI-C induced by TGF-β1 is mediated by the 26S proteasome.

### Smad3 is required for the degradation of NFI-C induced by TGF-β1

**Figure 3:**
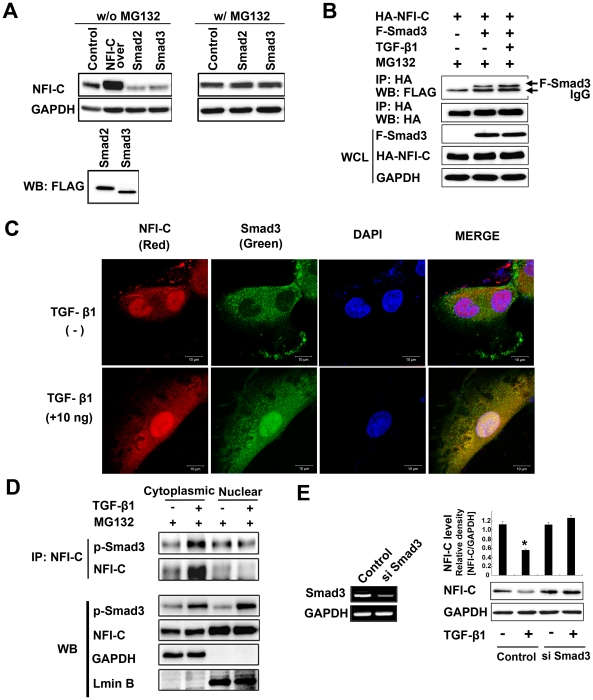
Degradation of NFI-C by TGF-β1 induction requires Smad3 in MDPC-23 cells. (**A**) MDPC-23 cells were transfected with empty vector (pCMV empty vector, control), Smad2, or Smad3 expression vectors in the presence or absence of MG132. NFI-C protein levels were analyzed by western blot 48 hr post-transfection. GAPDH was used as a loading control. (**B**) HEK293T cells were co-transfected with HA-tagged NFI-C and FLAG-tagged Smad3 (F-Smad3) expression vectors for 48 hr and then lysed. The whole cell lysates (WCL) and NFI-C immunoprecipitates were analyzed by western blot with anti-FLAG antibody. (**C**) Co-localization of NFI-C (red) and FLAG-Smad3 (green). MDPC-23 cells were co-transfected with NFI-C and FLAG-tagged Smad3 expression vector for 48 hr. After 48 hr, transfected cells were stimulated with TGF-β1 for 1 hr in the presence of MG132 (10 µM). Double immunostaining was performed with anti-NFI-C and anti-FLAG antibodies, followed by the secondary antibodies conjugated to goat anti-rabbit rhodamine (red, NFI-C) and goat anti-mouse FITC (green, FLAG-Smad3), respectively. The merged panel shows co-localization (yellow) of NFI-C and FLAG-Smad3 in the cytoplasm. DAPI staining was used to detect nuclei. (**D**) MDPC-23 cells were stimulated with TGF-β1 (10 ng/ml) in the presence of the proteasome inhibitor, MG132 (10 µM) for 1 hr. Cytoplasmic and nuclear fractions were isolated and NFI-C immunoprecipitates were analyzed by western blot with the anti-NFI-C or anti-pSmad3 antibody. GAPDH and lamin B served as cell fractionation controls. (E) MDPC-23 cells were transfected with shRNA Smad3 expression vector or control empty vector. Knockdown of Smad3 was evaluated by RT-PCR (left panel). Cell lysates were analyzed by western blot with NFI-C antibody, and the results were quantified using ImageJ (right panel; *P<0.01).

TGFβ-RI is recruited and phosphorylated upon binding of TGF-β1 to TGFβ-RII. Activated TGFβ-RI phosphorylates Smad2 and Smad3, forming a complex with a common partner, Smad4, and is subsequently translocated into the nucleus to regulate target gene transcription [18], [19], [20]. To determine whether Smad2 and Smad3 proteins have roles in the control of NFI-C protein levels, we measured both the protein and mRNA levels of NFI-C after either TGF-β1 treatment or overexpression of Smad2 and Smad3 in MDPC-23 cells. Endogenous NFI-C protein was degraded when either Smad2 or Smad3 was overexpressed in MDPC-23 cells but not when the proteasome inhibitor MG132 was used to block NFI-C degradation (Figure 3A). In contrast, *NFI-C* mRNA expression levels were increased after TGF-β1 treatment and Smad3 overexpression (Figure S3). These results suggest that the activation of TGF-β1 signaling resulted in the decrease of NFI-C protein levels, not via the inhibition of NFI-C transcription but by NFI-C protein degradation.

It was previously reported that Smad3, but not Smad2, binds to NFI-C through its MH1 domain [14]. Therefore, we looked for interactions between the Smads and NFI-C in immunoprecipitation assays. NFI-C directly interacted with Smad3 (Figure 3B), but not with Smad2 (data not shown). The amount of Smad3 binding to NFI-C was increased in the presence of TGF-β1 (Figure 3B). In the absence of TGF-β1, NFI-C was primarily localized to the nucleus, whereas Smad3 mainly localized to the cytoplasm of MDPC-23 cells. However, in the presence of TGF-β1, NFI-C and Smad3 proteins both localized to the nucleus and cytoplasm. Collectively, NFI-C and Smad3 proteins were co-localized to the cytoplasm after TGF-β1 treatment (Figure 3C). In the presence of MG132, TGF-β1 treatment increased the p-Smad3 in cytoplasm and nucleus. To determine whether NFI-C interacted with Smad3 in cytoplasm or nucleus, we examined endogenous NFI-C binding to p-Smad3 in immunoprecipitation assays in MDPC-23 cells. Endogenous NFI-C interacted with p-Smad3 in cytoplasm and nucleus of untreated cells. The amount of p-Smad3 binding to NFI-C after TGF-β1 treatment was increased in the cytoplasm but decreased in the nucleus (Figure 3D). To determine the effects of Smad3 on the TGF-β1-induced degradation of NFI-C, we measured the levels of NFI-C protein after siRNA-mediated Smad3 knockdown in MDPC-23 cells. Smad3 knockdown in MDPC-23 cells prevented the TGF-β1-induced degradation of NFI-C (Figure 3E). These results suggest that Smad3 mediate the degradation of NFI-C induced by TGF-β1.

### Ubiquitination and degradation of NFI-C by TGF-β1 is mediated by the ubiquitin ligases, Smurf1 and Smurf2

**Figure 4:**
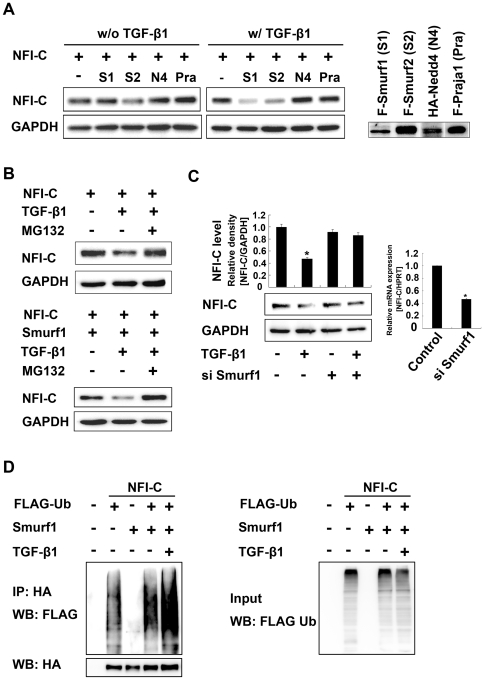
Ubiquitination and degradation of NFI-C by TGF-β1 is mediated by the ubiquitin ligase, Smurf1. (**A**) MDPC-23 cells were co-transfected with NFI-C, Smurf1, Smurf2, Nedd4, and Praja1 expression vectors for 48 hr. After 48 hr, transfected cells were incubated with or without TGF-β1 (10 ng/ml) for 1 hr. NFI-C protein levels were analyzed by western blot. GAPDH was used as a loading control. (**B**) MDPC-23 cells were co-transfected with NFI-C and Smurf1 expression vector for 48 hr. After 48 hr, transfected cells were incubated with or without TGF-β1 (10 ng/ml) in the presence or absence of MG132 for 1 hr. NFI-C protein levels were analyzed by western blot. (**C**) MDPC-23 cells were transfected with shRNA Smurf1 expression vector or control empty vector. Forty-eight hours post-transfection cells were stimulated with TGF-β1 for 1 hr. Cell lysates were analyzed by western blot with NFI-C antibody (left panel), and the results were quantified using ImageJ. Knockdown of Smurf1 was evaluated by real time PCR (right panel). (**D**) HEK293T cells were co-transfected with HA-tagged NFI-C, FLAG-tagged ubiquitin (Ub), and Smurf1, and then treated with MG132 (5 µM) for 48 hr. Forty-eight hours post-transfection, cells were stimulated with TGF-β1 for 1 hr. The NFI-C immunoprecipitates or whole cell lysates (WCL) were analyzed by western blot with anti-FLAG or anti-HA antibody.

E3 ubiquitin (Ub) ligases play key roles in the selective recognition of target proteins and subsequent protein degradation by the 26S proteasome [21]. To determine whether TGF-β1 activates the E3 Ub ligases during TGF-β1-dependent NFI-C degradation, we measured the ability of various E3 Ub ligases, including Smurf1, Smurf2, Nedd4, and Praja1, to ubiquitinate and degrade NFI-C in MDPC-23 cells. In the absence of TGF-β1, exogenous NFI-C was not degraded by either Smurf1 or Smurf2; however, in the presence of TGF-β1, both Smurf1 and Smurf2 significantly increased the degradation of NFI-C (Figure 4A). The proteasome inhibitor MG132 inhibited the degradation of NFI-C by Smurf1 and Smurf2 after the TGF-β1 stimulation of MDPC-23 cells (Figures 4B and S4A). In addition, Smurf1 knockdown also blocked the degradation of endogenous NFI-C in response to TGF-β1 (Figure 4C). We next asked whether either Smurf1 or Smurf2 induces the ubiquitination of NFI-C after treatment with TGF-β1. Although NFI-C showed weak polyubiquitination in the absence of Smurf1, Smurf2, and TGF-β1, polyubiquitination of NFI-C was increased by co-transfection of Ub with either Smurf1 or Smurf2 in HEK293T cells (Figures 4D and S4B). In particular, Smurf1 and Smurf2 markedly increased the polyubiquitination of NFI-C in the presence of TGF-β1 (Figures 4D and S4B). These results indicate that the degradation and ubiquitination of NFI-C induced by TGF-β1 signaling requires both Smurf1 and Smurf2.

### The interaction of NFI-C with Smurf1 and Smurf2 requires the activation of the MAP kinase pathway by TGF-β signaling

**Figure 5:**
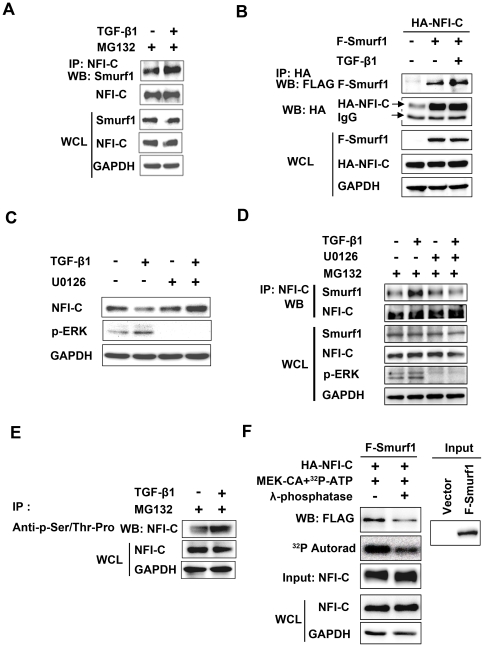
NFI-C interaction with Smurf1 requires activation of the MAPK pathway by TGF-β signaling. (**A**) MDPC-23 cells were treated with TGF-β1 (10 ng/ml) for 1 hr and then lysed. The NFI-C immunoprecipitates or whole cell lysates (WCL) were subjected to western blot analysis with the anti-Smurf1 or anti-NFI-C antibody. GAPDH was used as a loading control. (**B**) HEK293T cells were co-transfected with HA-tagged NFI-C and FLAG-tagged Smurf1 expression vectors for 48 hr. WCL and NFI-C immunoprecipitates were analyzed by western blot with anti-FLAG or anti-HA antibody. (**C**) MDPC-23 cells were incubated with TGF-β1 (1 hr, 10 ng/ml) in the presence or absence of the MEK inhibitor, U0126 (10 µM), added 1 hr prior to TGF-β1 addition. Cells were lysed, and NFI-C, p-ERK, and GAPDH levels were analyzed by western blot. (**D**) MDPC-23 cells were stimulated with TGF-β1 (10 ng/ml) for 1 hr in the presence or absence of the MEK inhibitor, U0126 (10 µM). WCL and NFI-C immunoprecipitates were analyzed by western blot. (**E**) WCL and anti-phospho-Ser/Thr-Pro immunoprecipitates were analyzed by western blot with anti-NFI-C antibody. (**F**) HA-tagged NFI-C protein was metabolically labeled with [γ-^32^P]-ATP in HEK293T cells. Bound Smurf1 proteins were eluted from the beads and detected by western blot analysis with the indicated antibody. The incorporation of ^32^P was detected by autoradiography, and the amount of HA-NFI-C was detected by western blot analysis.

To determine whether Smurf-NFI-C complexes are formed through a direct or indirect physical interaction, we examined endogenous NFI-C binding to Smurfs in immunoprecipitation assays in MDPC-23 cells. Endogenous NFI-C interacted with Smurf1 and Smurf2 in untreated cells. TGF-β1 treatment increased the interaction of endogenous NFI-C with both Smurf1 and Smurf2 (Figures 5A and S5A). However, fibroblast growth factor and epidermal growth factor treatment hardly influenced the interaction of endogenous NFI-C with both Smurf1 and Smurf2 (data not shown). We then performed immunoprecipitation assays using HEK293T cells overexpressing HA-tagged NFI-C and FLAG-tagged Smurf1 or Smurf2 proteins. NFI-C directly interacted with both Smurf1 and Smurf2, and TGF-β1 stimulation increased this binding (Figures 5B and S5B). These data indicate that NFI-C binds to Smurf1 and Smurf2 in a TGF-β1-dependent manner.

To determine whether the activation of mitogen-activated protein kinase (MAPK) by TGF-β1 is required for the degradation of NFI-C protein and the interaction between NFI-C and either Smurf1 or Smurf2, we treated MDPC-23 cells with the MEK inhibitor U0126 and analyzed the expression of NFI-C by western blot and immunoprecipitation. NFI-C was degraded by TGF-β1, but the addition of U0126 prevented the degradation of NFI-C in the presence of TGF-β1 (Figure 5C). Next, we asked whether endogenous NFI-C interaction with either Smurf1 or Smurf2 is affected by the presence of U0126 in MDPC-23 cells. NFI-C showed an increased interaction with both Smurf1 and Smurf2 induced by TGF-β1, whereas U0126 decreased NFI-C binding to both Smurf1 and Smurf2 (Figures 5D and S5C). To determine whether NFI-C was itself phosphorylated in response to TGF-β1 signaling, immunoprecipitates using anti-phospho-Ser/Thr-Pro antibody were immunoblotted with anti-NFI-C antibody. The phosphorylation of NFI-C was increased by TGF-β1 in MDPC-23 cells (Figure 5E) as well as in HEK293T cells after co-transfection with a MEK-CA expression plasmid containing the constitutively active form of MAPK-ERK kinase (Figure S6). To confirm whether NFI-C phosphorylation downstream of MAPK activation was implicated in the interaction between NFI-C and either Smurf1 or Smurf2, we performed a binding assay between NFI-C and Smurf1 or Smurf2 after dephosphorylation of phosphorylated NFI-C *in vitro*. The amount of Smurf1 or Smurf2 pulled down with NFI-C in co-immunoprecipitation assays was markedly decreased in cell lysates treated with λ-phosphatase (i.e., dephosphorylated NFI-C), compared to untreated control (phosphorylated NFI-C; Figures 5F and S5D). These results indicate that TGF-β1 and MAPK activation enhance the interaction and formation of a complex between NFI-C and Smurf1 or Smurf2, which is required for the ubiquitination and degradation of NFI-C.

### NFI-C affects dephosphorylation of p-Smad2/3

**Figure 6:**
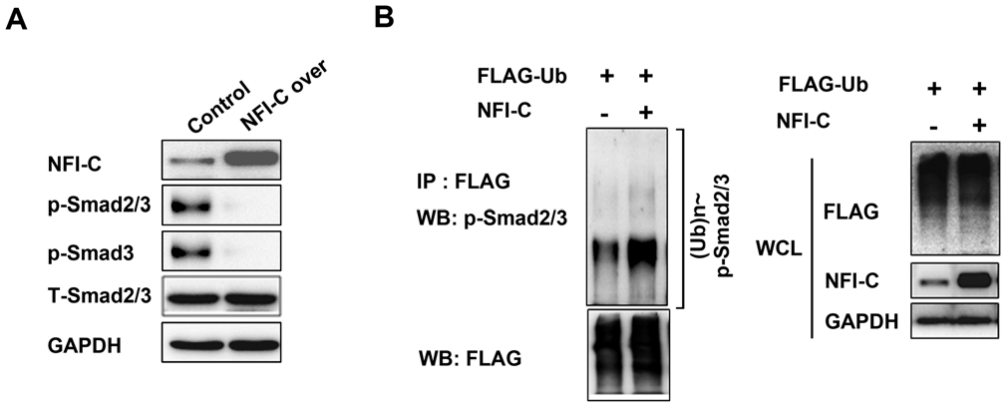
Dephosphorylation of p-Smad2/3 by NFI-C in MDPC-23 cells. (**A**) MDPC-23 cells were transfected with NFI-C expression vector for 48 hr. The level of NFI-C, p-Smad2/3, p-Smad3, and total Smad2/3 (T-Smad2/3) protein were analyzed by western blot. GAPDH was used as a loading control. (**B**) MDPC-23 cells were transfected with HA-tagged NFI-C and FLAG-tagged ubiquitin (Ub) expression vector for 48 hr. The FLAG immunoprecipitates or whole cell lysates (WCL) were analyzed by western blot.

To investigate whether NFI-C affects the expression of either Smad2/3 or Smad3 during *in vitro* odontoblast differentiation, we evaluated the effects of NFI-C overexpression on the levels of endogenous Smad2/3 protein by western blot. The results showed that NFI-C overexpression had no influence on the levels of total Smad2/3 protein but markedly decreased p-Smad2/3 and p-Smad3 protein levels, compared to untransfected control cells (Figure 6A). To determine whether the decrease in p-Smad2/3 protein levels observed after NFI-C overexpression was due to the degradation of p-Smad2/3 protein, we measured the polyubiquitination of p-Smad2/3 after overexpression of NFI-C in MDPC-23 cells. Polyubiquitination of endogenous p-Smad2/3 was hardly affected by NFI-C overexpression (Figure 6B). Based on these findings, we suggest that NFI-C overexpression resulted in a decrease in p-Smad2/3 protein levels, not via the degradation of p-Smad2/3 but the dephosphorylation of p-Smad2/3.

## Discussion

In *Nfic*-deficient mice, both the expression of TGF-β1 and TGFβ-RI, and the phosphorylation of Smad2/3 increase during odontoblast differentiation, leading to formation of abnormal dentin similar to osteodentin [15]. Mice in which NFI-C is disrupted are morphologically similar in terms of tooth dentin to TGF-β1-overexpressing transgenic mice [16]. The results of this study imply that a functional relationship exists between NFI-C and TGF-β1 signaling during odontoblast differentiation. Here, we investigated the mechanism of odontoblast differentiation by examining the mutual interaction between NFI-C and TGF-β1 signaling, and the degradation and ubiquitination of NFI-C by TGF-β1.

TGF-β1 induces cell growth arrest by activating p21, the cyclin-dependent kinase inhibitor, through the Smad signaling pathway [22]. Activation of p21 also induces keratinocyte differentiation [23]. Interestingly, NFI is an important factor in the inhibition of p21 expression in proliferating Hela cells [24]. In the present study, TGF-β1 induced the degradation of the NFI-C protein in both the cytoplasm and nucleus through the proteasome pathway, and NFI-C was degraded by the overexpression of Smad2 and Smad3. However, when Smad3 was inactivated by knockdown, TGF-β1 could not affect the degradation of NFI-C. Based on these findings, we suggest that TGF-β1 induces odontoblast differentiation through the Smad signaling pathway by increasing p21 and TGF-β-responsive gene expression levels via the degradation of NFI-C, which suppresses p21 expression.

In the present study, we showed that NFI-C interacts with Smad3 and is degraded in the cytoplasm and nucleus, which increases in response to stimulation with TGF-β1. Interestingly, the amount of Smad3 binding to NFI-C after TGF-β1 stimulation is increased in cytoplasm but decreased in the nucleus. Therefore, we suggest that in the cytoplasm, phosphorylated Smad3 induced by TGF-β1 stimulation increases binding to NFI-C, and results in the degradation of NFI-C.

NFI-C does not contain a polyproline-tyrosine motif, whereas a database search identified many SP sites and Ser/Thr protein kinase phosphorylation sites (SSXS), in the C-terminal region of NFI-C [13]. Recently, Smurf1 binding was shown to require the phosphorylation of SP sites located upstream of the PPAY motif in Smad1. MAPK-induced phosphorylation of the Smad1 linker region through bone morphogenetic protein signaling is recognized by Smurf1 and leads to the ubiquitination and degradation of Smad1 [25]. TGF-β1 also enhances the phosphorylation of the Smad2/3 linker regions by MAPK, which is involved with E3 Ub ligases, such as Nedd4L [26]. In the present study, we show that the interaction between NFI-C and Smurf1/Smurf2 were also required for the activation of MAPK-ERK by TGF-β1 signaling. Interestingly, TGF-β1-induced NFI-C binding to Smurf1 and Smurf2 was inhibited not by a combination of a p38 inhibitor but by a JNK inhibitor (data not shown). It suggests that JNK pathway is also involved in the interaction between NFI-C and Smurf1/Smurf2 induced by TGF-β1. Therefore, JNK pathway needs further investigation. Possible interaction motifs regulating the binding of NFI-C and either Smurf1 or Smurf2 are also currently under investigation.

When Smad signaling is continuously inhibited by the absence of TGF-β signaling, endogenous levels of Smad2 and Smad3 are negatively regulated by a number of proteins, including Smurfs. Smad2 and Smad3 show enhanced ubiquitination and degradation by Smurf2 and WW domain-containing protein 2 [27], [28]. In addition, the Axin and Ub ligase complex ROCK1 induces the degradation and ubiquitination of Smad3 [29], [30]. Therefore, we investigated whether the expression of Smad2/3 and/or Smad3 protein was affected by NFI-C in odontoblasts. NFI-C overexpression remarkably decreased the level of p-Smad2/3 and p-Smad3 protein via the dephosphorylation of p-Smad2/3 and p-Smad3 in MDPC-23 cells. These results suggest that NFI-C is a novel negative regulator of TGF-β1 signaling during odontoblast differentiation in tooth development. Recently, it was reported that NFI-C acts primarily to repress gene expression in response to TGF-β1 during wound healing [31]. This report has also provided evidence in support of our data.

To investigate whether the NFI-C interaction with TGF-β1 occurs in cell types other than odontoblasts, we measured the effects of TGF-β1 treatment on endogenous NFI-C protein levels in the human breast epithelial cell line, MCF10A. Expression of NFI-C was clearly observed in MCF10A cells. The level of NFI-C protein decreased in response to TGF-β1 treatment (Figure S7). These results demonstrate that degradation of NFI-C induced by TGF-β1 occurs generally in cell types other than odontoblasts in normal human breast epithelial cells.

**Figure 7:**
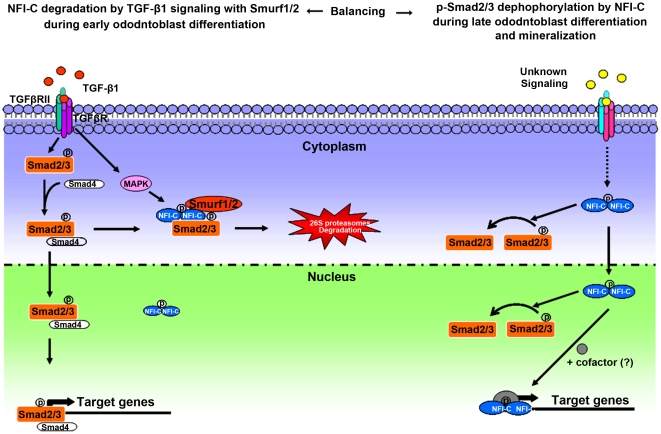
A proposed model of the function of NFI-C and TGF-β1 during odontoblast differentiation and homeostasis. During early odontoblast differentiation, p-Smad2/3 induced by TGF-β1 signaling increased binding to NFI-C in the cytoplasm. On the other hand, MAPK activation by TGF-β1 signaling increased the interaction of phosphorylated NFI-C and Smurf1/2. Collectively, TGF-β1 and MAPK activation enhanced the interaction and formation of Smad2/3-NFI-C-Smurf1/2 complex and resulted in the degradation of NFI-C. In contrast, during late odontoblast differentiation and mineralization, NFI-C signaling resulted in the dephosphorylation of p-Smad2/3. Consequently, TGF-β1 induces odontoblast differentiation through the Smad signaling pathway in early odontoblast differentiation, while NFI-C signaling modulates late odontoblast differentiation and mineralization.

In conclusion, as shown in Figure 7, these results demonstrate that interactions between NFI-C and TGF-β1 signaling play key roles in the regulation of odontoblast differentiation and homeostasis.

## Materials and Methods

### Antibodies

Antisera against NFI-C and DSP were produced as described previously [15]. Mouse monoclonal anti-hemagglutinin (HA; MMS-101P) and anti-FLAG (M2 and F3165) antibodies were purchased from COVANCE (Emeryville, CA) and Sigma-Aldrich (St Louis, MO), respectively. Rabbit anti-p-Smad3 and total Smad2/3 antibodies were purchased from Cell Signaling Technology (Danvers, MA). Mouse monoclonal anti-Runx-2 antibody was a gift from Dr. H-M Ryoo (Department of Cell and Developmental Biology, School of Dentistry, Seoul National University, Seoul, Korea). All other antibodies against TGFβ-RI (sc-398), TGFβ-RII (sc-400), p-Smad2/3 (sc-11769), p21 (sc-6246), and Osterix (sc-22538) were purchased from Santa Cruz Biotechnology (Santa Cruz, CA).

### Plasmid construction

pCH-NFIC, Samd2, and Smad3 expression plasmids were constructed, as described previously [15]. Smurf1, Smurf2, Nedd4, Praja1, TGFβ-RI (TD), MEK-CA, and FLAG-Ub plasmids were kind gifts from Dr. H-M Ryoo.

siRNAs were synthesized (Integrated DNA Technologies, San Diego, CA) based on the chosen 19 nucleotides of NFI-C (5′-CCG GTG AAG AAG ACA GAG A-3′), Smad3 (5′-ATC CGT ATG AGC TTC GTC A-3′), and Smurf1 (5′-GAA CCT TGC AAA GAA AGA C-3′), and these siRNA-expression plasmids were prepared using the pSUPER-retro-neo-GFP retro virus siRNA expression vector (OligoEngine, Seattle, WA), according to the manufacturer's instructions.

### Cell culture, transfection, and TGF-β1 stimulation

MDPC-23 (a generous gift from Dr. J. E. Nör, School of Dental Medicine, University of Michigan, MI), and HEK293T cells (ATCC, Rockville, MD) were grown and maintained in Dulbecco's modified Eagle's medium (DMEM, Gibco BRL, Carlsbad, CA) supplemented with 10% fetal bovine serum (FBS, Gibco BRL) and antibiotics (Penicillin-G 100 U/ml, streptomycin 100 µg/ml, fungizone 2.5 µg/ml, Gibco BRL) in 5% CO_2_ in a 37°C incubator. To induce MDPC-23 cell differentiation, 80–90% confluent cells were cultured in DMEM supplemented with 5% FBS, ascorbic acid (50 µg/ml), and β-glycerophosphate (10 mM) for up to 3 weeks.

The indicated expression plasmid (2 µg) was transiently transfected into MDPC-23 cells using Lipofectamine Plus™ reagent (Invitrogen) according to the manufacturer's instructions. MDPC-23 cells were stimulated with TGF-β1 (10 ng/ml, Invitrogen), TGF-β2 (10 ng/ml, PeproTech, Rocky Hill, NJ), or TGF-β3 (10 ng/ml, PeproTech) at 37°C for 1 hr. The chemical inhibitors, U0126 (ERK inhibitor, 10 µM, Calbiochem, La Jolla, CA), SB203580 (p38 inhibitor, 10 µM, Sigma-Aldrich), SP600125 (JNK inhibitor, 10 µM, Sigma-Aldrich), and MG132 (10 µM, Sigma-Aldrich) were added to MDPC-23 cells 1 hr prior to treatment with TGF-β1.

### Retroviral production and virus infection

Platinum-E cells were transfected with a pSUPER-retro-neo-GFP retrovirus vector that encodes NFI-C, Smad3, or Smurf1 siRNA, or empty vector using Lipofectamine Plus™ reagent (Invitrogen). After 48 hr, viral supernatants were harvested and filtered using a 0.45-µm syringe filter. MDPC-23 or MCF10A cells (ATCC) were infected with virus supernatant in the presence of Polybrene (6 µg/ml).

### Reverse transcription (RT)-PCR and real-time PCR analysis

Total RNA was extracted from the MDPC-23 cells with TRIzol® reagent according to the manufacturer's instructions (Invitrogen). Total RNA (2 µg) was subjected to reverse transcription with 0.5 µg Oligo dT and 1 µl (50 IU) Superscript III enzyme (Invitrogen) in a 20-µl reaction mixture at 50°C for 1 hr. The resulting mixture was amplified by PCR. One microliter of the reverse transcription product was subjected to PCR: 32 cycles of 94°C for 0.5 min, 55°C for 0.5 min, 72°C for 0.5 min. For RT-PCR, specific primers for *DMP-1*, *ColIa1*, *Smad3*, and *GAPDH* were synthesized as listed in Table S1. The PCR products were electrophoresed on a 1.2% agarose gel, stained with ethidium bromide, and visualized under ultraviolet light.

For real-time PCR, specific primers for *NFI-C*, *DSPP*, *osteocalcin*, *Smad3, Smurf1*, and *HPRT* were synthesized as listed in Table S2. Real-time PCR was performed on an ABI PRISM 7500 sequence detection system with SYBR GREEN PCR Master Mix (Applied Biosystems, Foster City, CA) according to the manufacturer's instructions. The PCR conditions were 94°C for 1 min followed by 95°C for 15 s and 60°C for 34 s for 40 cycles. All reactions were run in triplicate and were normalized to the housekeeping gene, *HPRT*. The relative differences in PCR results were calculated using the comparative cycle threshold (C_T_) method.

### Western blot analysis and immunoprecipitation

Western blot analysis was performed as previously described [15]. Briefly, the proteins (30 µg) were separated by 10% sodium dodecyl sulfate-polyacrylamide gel electrophoresis (SDS-PAGE), transferred onto a nitrocellulose membrane (Schleicher & Schuell BioScience, Dassel, Germany), and labeled with specific antibodies. Labeled protein bands were detected using an enhanced chemiluminescence system (GE Healthcare, Buckinghamshire, UK), and bands were measured by densitometric analysis.

To prepare nuclear extracts, cells were resuspended in a hypotonic solution (10 mM HEPES, 10 mM KCl, and protease inhibitors) and incubated for 10 min on ice. Nonyl phenoxypolyethoxylethanol (NP-40) was added to a final concentration of 0.1%, and the nuclei were isolated by centrifugation at 1,000× *g* for 10 min at 4°C. Nuclei were washed three times with the same hypertonic solution. After removal of the supernatant, nuclei were resuspended in a nuclear lysis solution (10 mM HEPES, pH 7.5, 150 mM NaCl, 1% NP-40, 0.25% Na-deoxycholate, 10% glycerol, and protease inhibitors) and centrifuged at 16,000× *g* for 45 min at 4°C. For immunoprecipitation analysis, 500 µg precleared cell extracts were incubated with anti-HA antibody at 4°C overnight with constant rotation and then incubated at 4°C for 3 hr with 20 µl Protein A/G beads (Santa Cruz Biotechnology). The beads were washed extensively five times, resuspended in 2× Laemmli sample buffer, and boiled at 100°C for 5 min. Samples were resolved by 10% SDS-PAGE and analyzed by western blot using anti-FLAG and anti-HA antibodies. Densitometry was performed using ImageJ software (http://rsb.info.nih.gov/ij).

### Ubiquitination assay

Transfected HEK293T cells were incubated with 10 µM MG132 (Sigma-Aldrich). For the detection of recombinant Ub, cells were co-transfected with pFLAG-Ub and an expression vector pCH-NFI-C (tagged-HA). For immunoprecipitation analysis, cells were lysed in 100 µl TBS buffer (20 mM Tris, pH 7.5, 150 mM NaCl, and 2% SDS), boiled for 15 min, and briefly sonicated. The lysates were diluted with 900 µl TNX dilution buffer (20 mM Tris, pH 7.5, 150 mM NaCl, and 1% TritonX-100) and centrifuged at 16,000× *g* at 4°C for 45 min. Cleared lysate (1 mg) was subjected to immunoprecipitation with the anti-HA antibody. Enriched Ub-conjugated proteins were resolved by 8% SDS-PAGE and analyzed by western blot using anti-HA or anti-FLAG antibodies.

### Kinase assay and in vitro binding assay

HEK293T cells were co-transfected with NFI-C and MEK-CA expression plasmids, and cultured for 24 hr. Labeling was conducted for 2 hr in DMEM-0.1% FBS medium containing 100 µCi [γ-^32^P]-adenosine triphosphate (ATP; PerkinElmer Life Sciences, Inc. Waltham, MA). Whole cell lysates were prepared as previously described [15]. Pre-cleared cell extracts were incubated with anti-HA antibody at 4°C overnight with constant rotation, and then incubated at 4°C for 3 hr with 20 µl Protein A/G beads (Santa Cruz Biotechnology). The beads were washed five times. Purified HA-NFI-C protein A/G beads were incubated with or without lambda protein phosphatase (New England Biolabs, Ipswich, MA) at 30°C for 1 hr. For interaction assay, beads were incubated with HEK293T lysates expressing FLAG-tagged Smurf1 and Smurf2 proteins at 4°C for 2 hr. The beads were washed twice and boiled in Laemmli sample buffer for western blot analysis. The incorporation of ^32^P into ERK-phosphorylated HA-NFI-C was detected by autoradiography.

### Immunofluorescence microscopy

MDPC-23 cells were cultured on Laboratory-Tek chambered coverglasses (Nunc, Rochester, NY) and stimulated with TGF-β1 (10 ng/ml, Invitrogen) at 37°C for 1 hr. The chemical inhibitor MG132 (10 µM, Sigma-Aldrich) was added to MDPC-23 cells 1 hr prior to treatment with TGF-β1. For fluorescence microscopy, the cells were fixed with 4% paraformaldehyde in PBS at 4°C for 20 min, permeabilized with 0.15% Triton X-100 in PBS for 10 min, and then pre-incubated with 1% bovine serum albumin in PBS for 30 min. Cells were incubated for 2 hr with rabbit-anti-NFI-C antibody (1∶100), washed three times with PBS, and then incubated for 45 min with a fluorescent secondary antibody (rabbit-anti-IgG-FITC, 1∶500, Invitrogen) and counterstained with 4′-6-diamidino-2-phenylindole (DAPI). Cells were examined by confocal laser scanning microscopy (Olympus, Tokyo, Japan).

### Statistical analysis

Data were analyzed for statistical significance using a non-parametric Mann-Whitney test.

## Supporting Information

Figure S1 **The mRNA expression levels of** ***osteocalcin*** **,** ***DSPP*** **,** ***ColIa1*** **, and** ***DMP-1*** **during odontoblast differentiation.** Expression of *OC* (**A**) and *DSPP* (**B**) mRNA analyzed by real-time PCR. Expression of *ColIa1* (**C**) and *DMP-1* (**D**) mRNA analyzed by RT-PCR. The results were quantified using ImageJ. Data are presented as the mean ± standard deviation (SD) for three separate experiments. An asterisk denotes values significantly different from the control (0 day) using a nonparametric Mann-Whitney test (* P<0.01).
(TIF)

Figure S2 **NFI-C is degraded by TGF-β1 in MDPC-23 cells.** (**A**) MDPC-23 cells were incubated with TGF-β1 (10 ng/ml), TGF-β2 (10 ng/ml), and TGF-β3 (10 ng/ml) for 1 hr. NFI-C protein levels were analyzed by western blot. GAPDH was used as a loading control. (**B**) MDPC-23 cells were transfected with empty vector (pCMV empty vector, control) or TGFβ-RI. TGFβ-RI and NFI-C protein levels were analyzed by western blot 48 hr post-transfection. GAPDH was used as a loading control. (**C**) MDPC-23 cells were treated with 0.1, 1, 5, 10, or 100 ng/ml TGF-β1 for 1 hr. NFI-C protein levels were analyzed by western blot.
(TIF)

Figure S3 **Effects of NFI-C, Smad3, and TGF-β1 on mRNA expression levels of** ***NFI-C*** **and** ***Smad3*** **in MDPC-23 cells.** MDPC-23 cells were transfected with NFI-C, Smad3, siRNA NFI-C, and siRNA Smad3 expression vector or control empty vector for 48 hr, and treated with TGF-β1 (10 ng/ml). Expression of (**A**) *NFI-C* and (**B**) *Smad3* mRNA were analyzed by real-time PCR.
(TIF)

Figure S4 **Ubiquitination and degradation of NFI-C by TGF-β1 is mediated by the ubiquitin ligase, Smurf2.** (**A**) MDPC-23 cells were co-transfected with NFI-C and Smurf2 expression vector for 48 hr. Forty-eight hours post-transfection, cells were incubated with or without TGF-β1 (10 ng/ml) in the presence or absence of MG132 for 1 hr. NFI-C protein levels were analyzed by western blot. GAPDH was used as a loading control. (**B**) HEK293T cells were co-transfected with HA-tagged NFI-C, FLAG-tagged ubiquitin (Ub), and Smurf2 and treated MG132 (5 µM) for 48 hr. After 48 hr, transfected cells were stimulated with TGF-β1 for 1 hr. The NFI-C immunoprecipitates (left panel) or whole cell lysates (right panel) were analyzed by western blot with anti-FLAG or anti-HA antibody.
(TIF)

Figure S5 **NFI-C interaction with Smurf2 requires the activation of the MAPK pathway by TGF-β signaling.** (**A**) MDPC-23 cells were treated with TGF-β1 (10 ng/ml) for 1 hr and then lysed. The NFI-C immunoprecipitates or whole cell lysates (WCL) were subjected to western blot analysis with the anti-Smurf2 or anti-NFI-C antibody. GAPDH was used as a loading control. (**B**) HEK293T cells were co-transfected with HA-tagged NFI-C and FLAG-tagged Smurf2 expression vectors for 48 hr. Cells were incubated with TGF-β1 (10 ng/ml) for 1 hr. WCL and NFI-C immunoprecipitates were analyzed by western blot with anti-FLAG or anti-HA antibody. (**C**) MDPC-23 cells were stimulated with TGF-β1 (10 ng/ml) for 1 hr in the presence or absence of the MEK inhibitor, U0126 (10 µM). WCL and NFI-C immunoprecipitates were analyzed by western blot. (**D**) HA-tagged NFI-C protein was metabolically labeled with [γ-^32^P]-ATP in HEK293T cells. Phosphorylated HA-NFI-C was incubated with or without λ phosphatase at 30°C for 1 hr. Phosphorylated and dephosphorylated HA-NFI-C was incubated with HEK293T lysates expressing the FLAG-tagged Smurf2 for 2 hr at 4°C. Bound Smurf2 proteins were eluted from the beads and detected by western blot analysis with the indicated antibody. The incorporation of ^32^P was detected by autoradiography, and the amount of HA-NFI-C was detected by western blot analysis.
(TIF)

Figure S6 **Phosphorylation of NFI-C is increased by the activation of MAPK.** HEK293T cells were co-transfected with HA-tagged NFI-C and MEK-CA expression vectors for 48 hr. Whole cell lysates and anti-phospho-Ser/Thr-Pro immunoprecipitates were analyzed by western blot with anti-HA antibody.
(TIF)

Figure S7 **NFI-C is degraded by TGF-β1 in normal human breast epithelial cells.** Normal human breast epithelial cells (MCF-10A cells) were cultured in DMEM/F12 supplemented with 5% horse serum, insulin (0.01 mg/ml), EGF (20 ng/ml), cholera toxin (100 ng/ml), hydrocortisone (500 ng/ml), 2 mM L-glutamine, and antibiotics. Cells were infected with retroviral supernatant containing NFI-C siRNA and/or NFI-C for overexpression or empty vector, and treated with TGF-β1 (10 ng/ml) for 1 hr. NFI-C protein levels were measured by western blot analysis (left panel), and the results were quantified using ImageJ (right panel). GAPDH was used as a loading control.
(TIF)

Table S1 **Nucleotide sequences of RT- PCR primer pairs.**
(DOC)

Table S2 **Nucleotide sequences of real-time PCR primer pairs.**
(DOC)
