# Supplementary figures and images for: Crosstalk between Nuclear Factor I-C and Transforming Growth Factor-β1 Signaling Regulates Odontoblast Differentiation and Homeostasis
Source: PLoS One. 2011 Dec 16;6(12):e29160. doi: 10.1371/journal.pone.0029160 (PMC3241690; doi:10.1371/journal.pone.0029160)

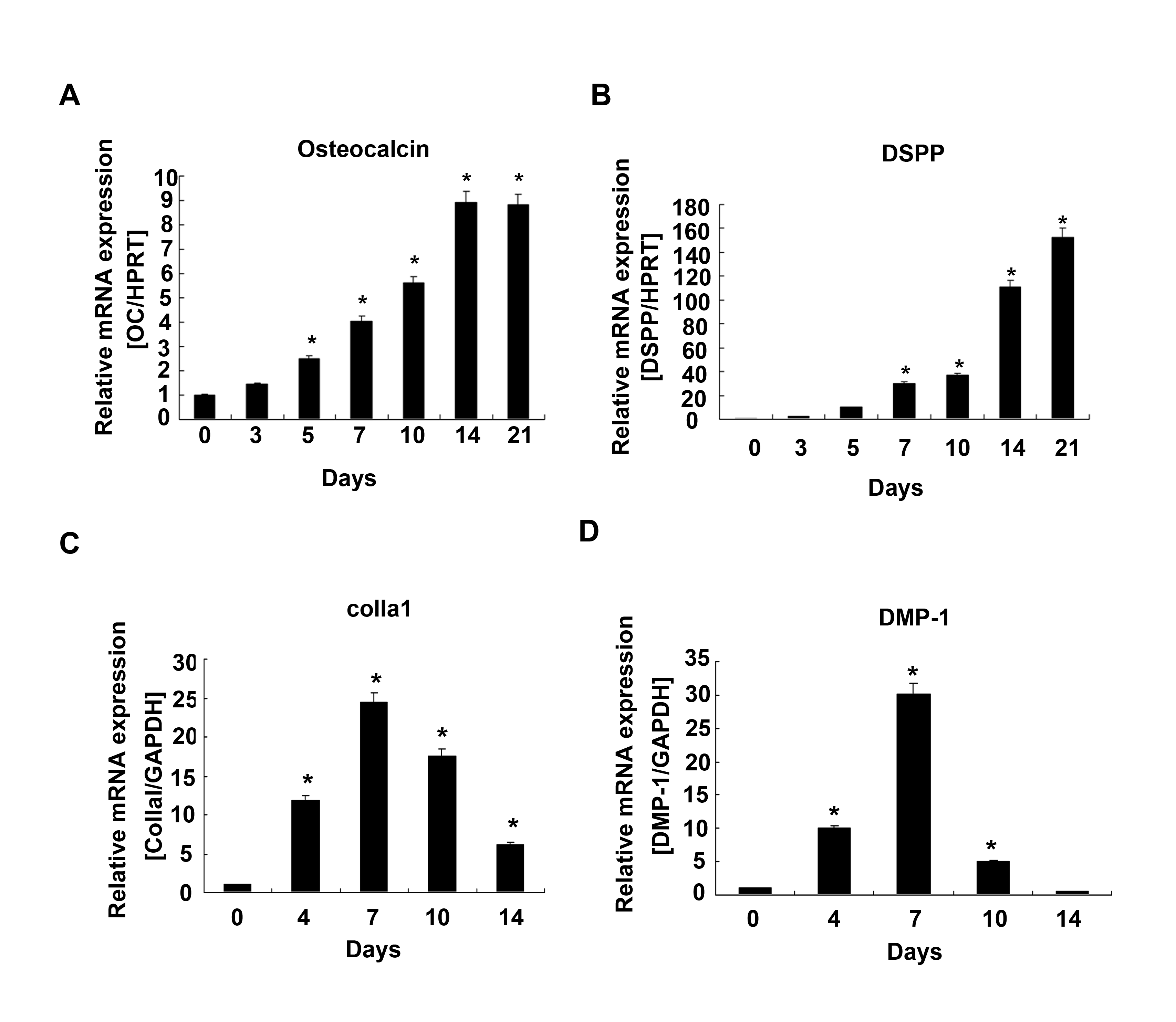

Supplement: Figure S1 — The mRNA expression levels of osteocalcin , DSPP , ColIa1 , and DMP-1 during odontoblast differentiation. Expression of OC (A) and DSPP (B) mRNA analyzed by real-time PCR. Expression of ColIa1 (C) and DMP-1 (D) mRNA analyzed by RT-PCR. The results were quantified using ImageJ. Data are presented as the mean ± standard deviation (SD) for three separate experiments. An asterisk denotes values significantly different from the control (0 day) using a nonparametric Mann-Whitney test (* P<0.01). (TIF) [file pone.0029160.s001.tif]

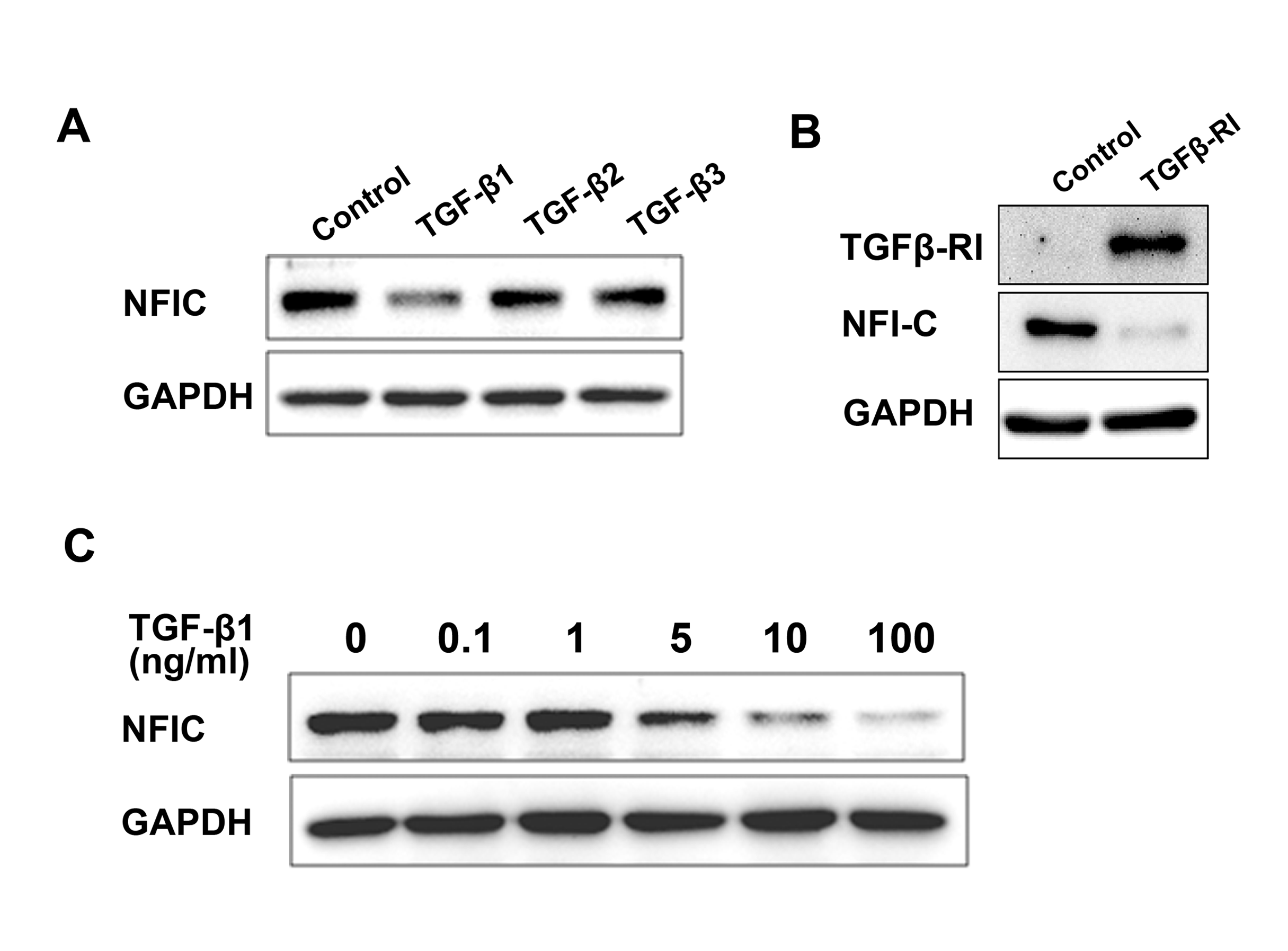

Supplement: Figure S2 — NFI-C is degraded by TGF-β1 in MDPC-23 cells. (A) MDPC-23 cells were incubated with TGF-β1 (10 ng/ml), TGF-β2 (10 ng/ml), and TGF-β3 (10 ng/ml) for 1 hr. NFI-C protein levels were analyzed by western blot. GAPDH was used as a loading control. (B) MDPC-23 cells were transfected with empty vector (pCMV empty vector, control) or TGFβ-RI. TGFβ-RI and NFI-C protein levels were analyzed by western blot 48 hr post-transfection. GAPDH was used as a loading control. (C) MDPC-23 cells were treated with 0.1, 1, 5, 10, or 100 ng/ml TGF-β1 for 1 hr. NFI-C protein levels were analyzed by western blot. (TIF) [file pone.0029160.s002.tif]

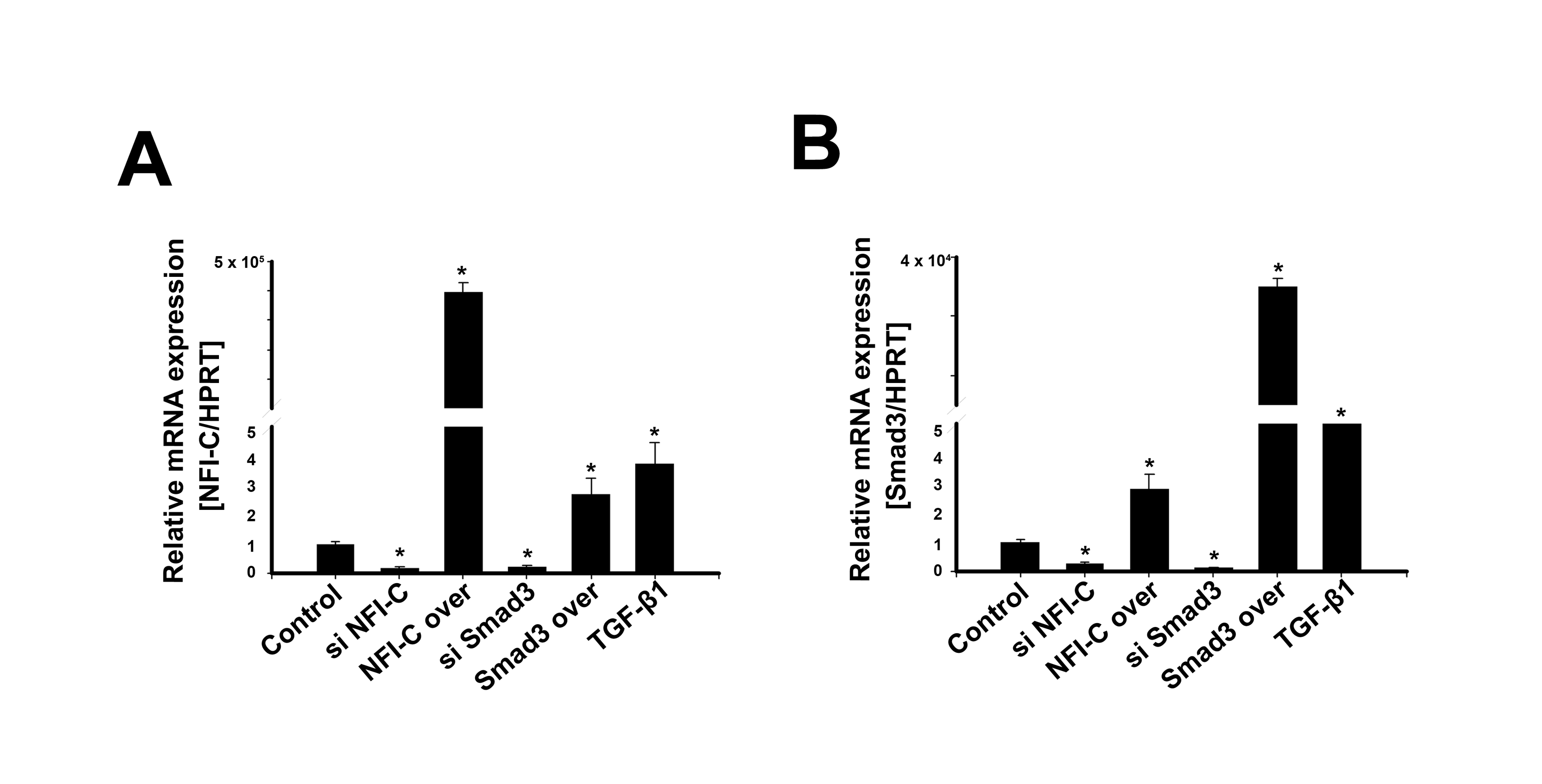

Supplement: Figure S3 — Effects of NFI-C, Smad3, and TGF-β1 on mRNA expression levels of NFI-C and Smad3 in MDPC-23 cells. MDPC-23 cells were transfected with NFI-C, Smad3, siRNA NFI-C, and siRNA Smad3 expression vector or control empty vector for 48 hr, and treated with TGF-β1 (10 ng/ml). Expression of (A) NFI-C and (B) Smad3 mRNA were analyzed by real-time PCR. (TIF) [file pone.0029160.s003.tif]

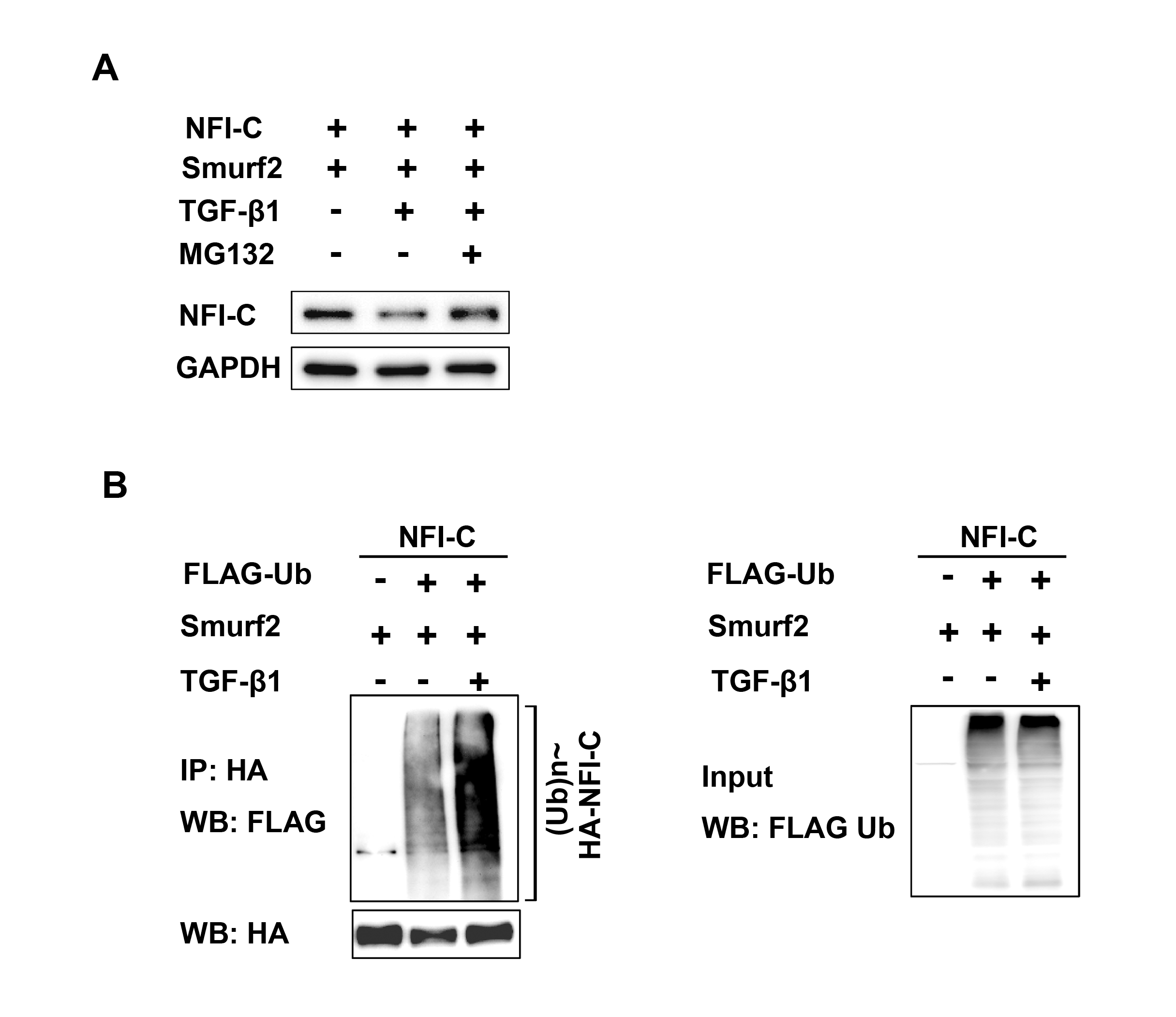

Supplement: Figure S4 — Ubiquitination and degradation of NFI-C by TGF-β1 is mediated by the ubiquitin ligase, Smurf2. (A) MDPC-23 cells were co-transfected with NFI-C and Smurf2 expression vector for 48 hr. Forty-eight hours post-transfection, cells were incubated with or without TGF-β1 (10 ng/ml) in the presence or absence of MG132 for 1 hr. NFI-C protein levels were analyzed by western blot. GAPDH was used as a loading control. (B) HEK293T cells were co-transfected with HA-tagged NFI-C, FLAG-tagged ubiquitin (Ub), and Smurf2 and treated MG132 (5 µM) for 48 hr. After 48 hr, transfected cells were stimulated with TGF-β1 for 1 hr. The NFI-C immunoprecipitates (left panel) or whole cell lysates (right panel) were analyzed by western blot with anti-FLAG or anti-HA antibody. (TIF) [file pone.0029160.s004.tif]

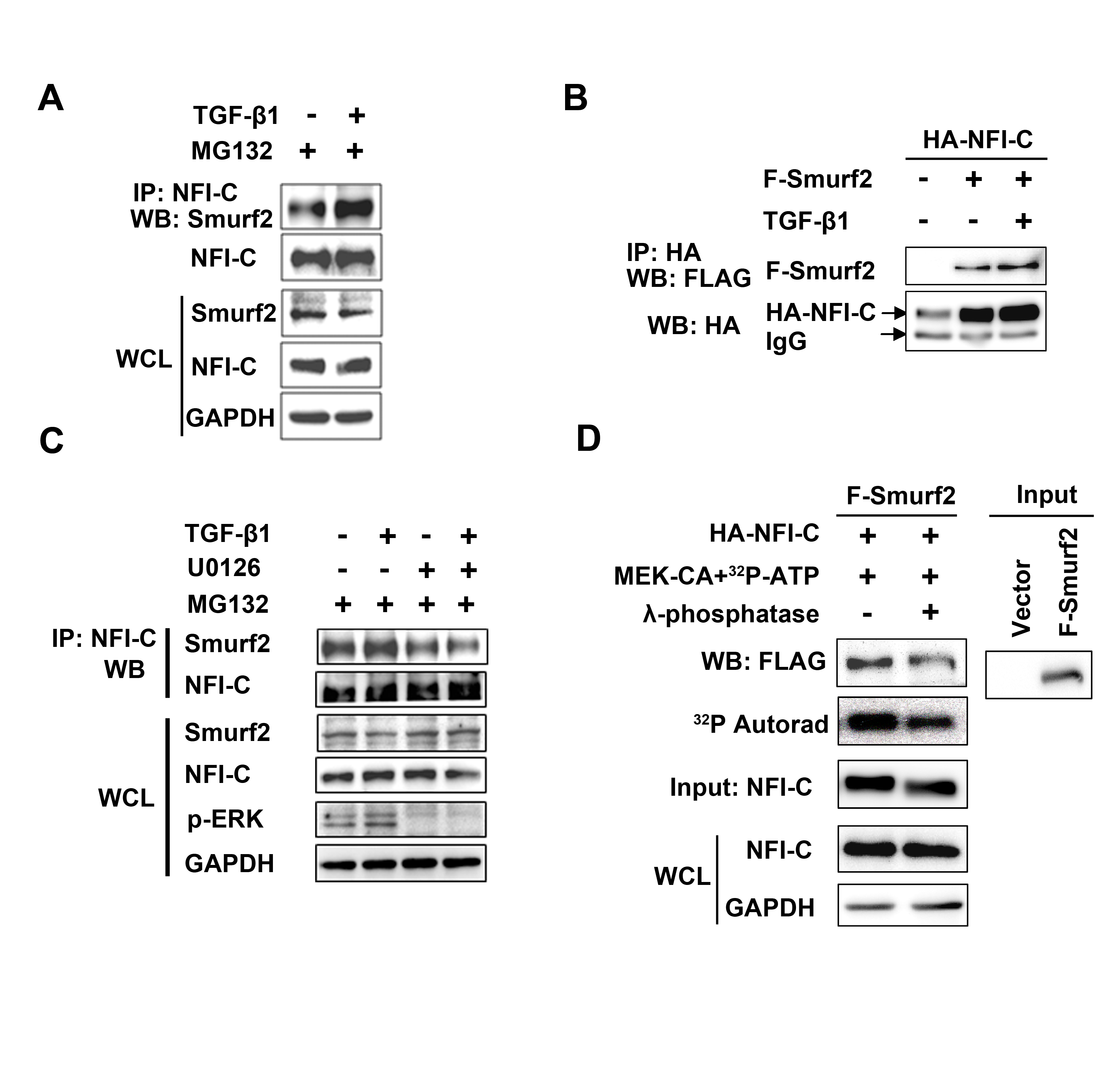

Supplement: Figure S5 — NFI-C interaction with Smurf2 requires the activation of the MAPK pathway by TGF-β signaling. (A) MDPC-23 cells were treated with TGF-β1 (10 ng/ml) for 1 hr and then lysed. The NFI-C immunoprecipitates or whole cell lysates (WCL) were subjected to western blot analysis with the anti-Smurf2 or anti-NFI-C antibody. GAPDH was used as a loading control. (B) HEK293T cells were co-transfected with HA-tagged NFI-C and FLAG-tagged Smurf2 expression vectors for 48 hr. Cells were incubated with TGF-β1 (10 ng/ml) for 1 hr. WCL and NFI-C immunoprecipitates were analyzed by western blot with anti-FLAG or anti-HA antibody. (C) MDPC-23 cells were stimulated with TGF-β1 (10 ng/ml) for 1 hr in the presence or absence of the MEK inhibitor, U0126 (10 µM). WCL and NFI-C immunoprecipitates were analyzed by western blot. (D) HA-tagged NFI-C protein was metabolically labeled with [γ-32P]-ATP in HEK293T cells. Phosphorylated HA-NFI-C was incubated with or without λ phosphatase at 30°C for 1 hr. Phosphorylated and dephosphorylated HA-NFI-C was incubated with HEK293T lysates expressing the FLAG-tagged Smurf2 for 2 hr at 4°C. Bound Smurf2 proteins were eluted from the beads and detected by western blot analysis with the indicated antibody. The incorporation of 32P was detected by autoradiography, and the amount of HA-NFI-C was detected by western blot analysis. (TIF) [file pone.0029160.s005.tif]

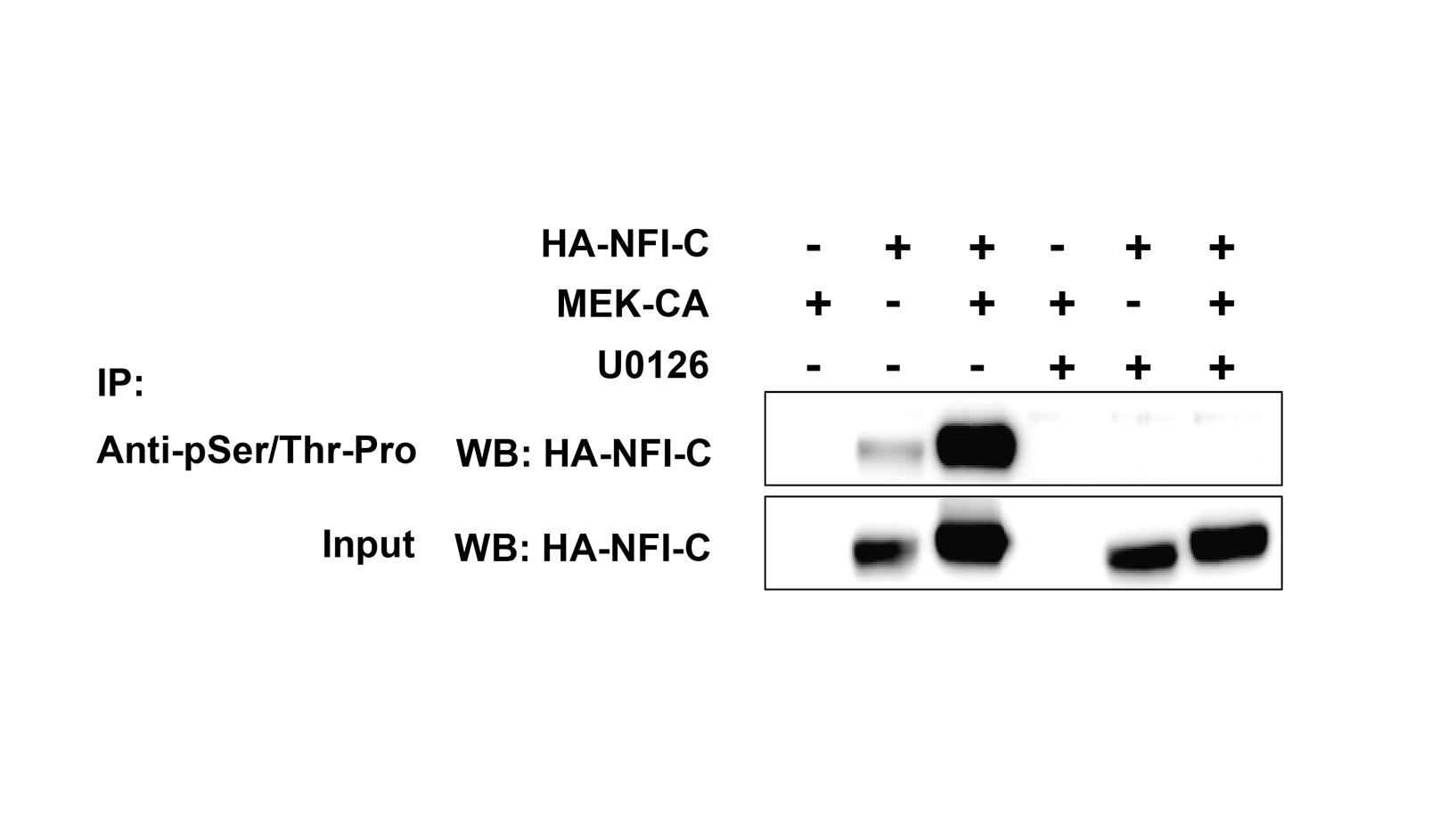

Supplement: Figure S6 — Phosphorylation of NFI-C is increased by the activation of MAPK. HEK293T cells were co-transfected with HA-tagged NFI-C and MEK-CA expression vectors for 48 hr. Whole cell lysates and anti-phospho-Ser/Thr-Pro immunoprecipitates were analyzed by western blot with anti-HA antibody. (TIF) [file pone.0029160.s006.tif]

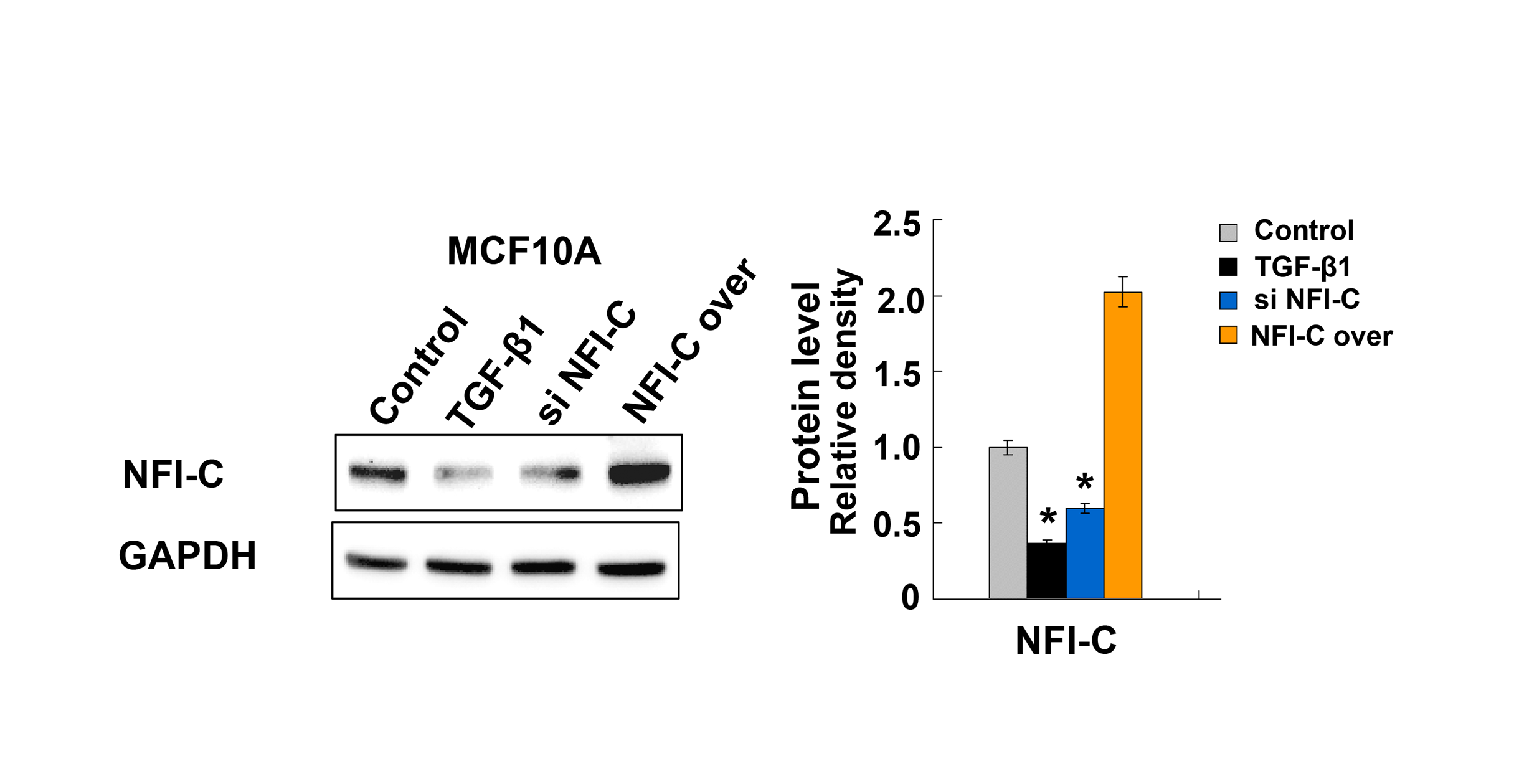

Supplement: Figure S7 — NFI-C is degraded by TGF-β1 in normal human breast epithelial cells. Normal human breast epithelial cells (MCF-10A cells) were cultured in DMEM/F12 supplemented with 5% horse serum, insulin (0.01 mg/ml), EGF (20 ng/ml), cholera toxin (100 ng/ml), hydrocortisone (500 ng/ml), 2 mM L-glutamine, and antibiotics. Cells were infected with retroviral supernatant containing NFI-C siRNA and/or NFI-C for overexpression or empty vector, and treated with TGF-β1 (10 ng/ml) for 1 hr. NFI-C protein levels were measured by western blot analysis (left panel), and the results were quantified using ImageJ (right panel). GAPDH was used as a loading control. (TIF) [file pone.0029160.s007.tif]
