# Supplementary material for: Crosstalk between Nuclear Factor I-C and Transforming Growth Factor-β1 Signaling Regulates Odontoblast Differentiation and Homeostasis
Source: PLoS One. 2011 Dec 16;6(12):e29160. doi: 10.1371/journal.pone.0029160 (PMC3241690; doi:10.1371/journal.pone.0029160)
Supplement: Table S1 — Nucleotide sequences of RT- PCR primer pairs. (DOC) [file pone.0029160.s008.doc]

**Table S1. Nucleotide sequences of RT-PCR primer pairs.**

| Gene | Primer (5'-3') | |
| --- | --- | --- |
| ColIa1 | forward | TAA GTT GCC AAG AAC GTG CC |
|  | reverse | AAT TGA AAG CCA GGA GGC AT |
| DMP-1 | forward | CGGCTGGTGGTCTCTCTAAG |
|  | reverse | ATCTTCCTGGGACTGGGTCT |
| Smad3 | forward | GAGTAGAGACGCCAGTTCTACC |
|  | reverse | GGTTTGGAGAACCTGCGTCCA |
| GAPDH | forward | ACCACAGTCCATGCCATCAC |
|  | reverse | TCCACCACCCTGTTGCTGT |
