# Supplementary material for: Crosstalk between Nuclear Factor I-C and Transforming Growth Factor-β1 Signaling Regulates Odontoblast Differentiation and Homeostasis
Source: PLoS One. 2011 Dec 16;6(12):e29160. doi: 10.1371/journal.pone.0029160 (PMC3241690; doi:10.1371/journal.pone.0029160)
Supplement: Table S2 — Nucleotide sequences of real-time PCR primer pairs. (DOC) [file pone.0029160.s009.doc]

**Table S2. Nucleotide sequences of real-time PCR primer pairs.**

| Gene | Primer (5'-3') | |
| --- | --- | --- |
| NFI-C | forward | GACCTGTACCTGGCCTACTTTG |
|  | reverse | CACACCTGACGTGACAAAGCTC |
| DSPP | forward | ATTCCGGTTCCCCAGTTAGTA |
|  | reverse | CTGTTGCTAGTGGTGCTGTT |
| OC | forward | CTGACAAAGCCTTCATGTCCAA |
|  | reverse | GCGCCGGAGTCTGTTCACTA |
| Smad3 | forward | CACGCAGAACGTGAACACC |
|  | reverse | GGCAGTAGATAACGTGAGGGA |
| Smurf1 | forward | AGTTCGTGGCCAAATAGTGG |
|  | reverse | GTTCCTTCGTTCTCCAGCAG |
| HPRT | forward | CCTAAGATGAGCGCAAGTTGAA |
|  | reverse | CCACAGGGACTAGAACACCTGCTA |
